# Management of Autoimmune urticaria in a patient with Myasthenia Gravis, a challenging case

**DOI:** 10.5339/qmj.2023.sqac.6

**Published:** 2023-11-19

**Authors:** Asaad Omer Imameldin, Sami Aqel, Ahmad Shihab Altabouki, Tayseer Ibrahim

**Affiliations:** ^1^Adult Allergy and Immunology Section, Department of Medicine, Hamad Medical Corporation, Doha, Qatar. Email: AImameldin@hamad.qa; ^2^Neurology Division, Department of Medicine, Hamad Medical Corporation, Doha, Qatar.

## Abstract

Introduction: The pathophysiology of chronic spontaneous urticaria (CSU) is not yet fully understood; however, increasing evidence supports the association between CSU and autoimmunity.

Myasthenia gravis (MG) is an autoimmune neuromuscular disorder. MG management relies on using immunosuppressants and avoiding certain medications that can precipitate an MG crisis.

The coexistence of CSU and MG was described in the literature on elderly patients. Herein we present a challenging case regarding the management of CSU in a young female with MG.

Case report: A 22-year-old lady known to have Myasthenia gravis post thymectomy, with a history of multiple MG crises, presented to the Allergy Clinic with recurrent itchy hives typical for urticaria without associated angioedema. Despite being on Azathioprine and low-dose steroids for MG treatment, she had an active CSU disease, UAS7:32, UCT: 5. The neurologist advised against the use of regular oral antihistamines because they might exacerbate MG. Although we do not have serum autologous skin testing, basophil activation test, or IgG autoantibodies for a definitive definition of autoimmune CSU (aiCSU), the patient has some features supporting the diagnosis of aiCSU (see Table 1). In addition, she has a normal total IgE level, normal C3, C4, negative RF, ANA, ANCA, anti-TPO, and anti-thyroglobulin antibodies.

After the discussion with the neurologist, we started her on Omalizumab 300 mg every four weeks, which was increased to 450 mg every two weeks with partial control of CSU. She was started on rituximab to treat MG with improvement in CSU.

Conclusion: To the best of our knowledge, limited data describing the association between MG and CSU in young patients. Moreover, there is insufficient data on the safety of antihistamines in patients with MG, which are the first line of treatment for CSU.

There are clinical and laboratory biomarkers that help in identifying CSU endotypes. Recognition of aiCSU endotype is essential as it helps predict disease course and response to treatment. Moreover, careful therapeutic interventions in patients with CSU and coexistent autoimmune diseases are warranted to achieve efficacy and reduce drug interactions and adverse effects.

## Figures and Tables

**Table 1.** Patient characteristics support Type IIb autoimmune CSU (aiCSU).

**Characteristics**
Female gender
Nocturnal symptoms
Presence of Myasthenia Gravis
Low eosinophil count (0.02)
Low IgA level (1.56 gm/L)
Low disease control
Severe disease
Poor response to Omalizumab

